# NINFA: Non-commercial interface for neuro-feedback acquisitions

**DOI:** 10.1117/1.NPh.12.2.026601

**Published:** 2025-05-14

**Authors:** Costanza Iester, Clint Banzhaf, Ahmed Eldably, Betti Schopp, Andreas J. Fallgatter, Laura Bonzano, Marco Bove, Ann-Christine Ehlis, Beatrix Barth

**Affiliations:** a University of Genoa, Department of Neuroscience, Rehabilitation, Ophthalmology, Genetics, Maternal and Child Health, Genoa, Italy; b Independent Researcher, Tübingen, Germany; c University Hospital, Tübingen Center for Mental Health, Tübingen Department of Psychiatry and Psychotherapy, Tübingen, Germany; d German Center for Mental Health, Partner site Tübingen, Germany; e University of Tübingen, LEAD Graduate School and Research Network, Tübingen, Germany; f IRCCS Ospedale Policlinico San Martino, Genoa, Italy; g University of Genoa, Section of Human Physiology, Department of Experimental Medicine, Genoa, Italy

**Keywords:** neurofeedback, real-time application, functional near-infrared spectroscopy

## Abstract

**Significance:**

In recent years, functional near-infrared spectroscopy (fNIRS) has gained increasing attention in the field of neurofeedback. However, there is a lack of freely accessible tools for research in this area that reflect the state of the art in research and technology.

**Aim:**

To address this need, we introduce Non-commercial Interface for Neuro-Feedback Acquisitions (NINFA), a user-friendly and flexible freely available neurofeedback application for real-time fNIRS, which is also open to other modalities such as electroencephalography (EEG).

**Approach:**

NINFA was developed in MATLAB and the lab streaming layer connection offers maximum flexibility in terms of combination with different fNIRS or EEG acquisition software and hardware.

**Results:**

The user-friendly interface allows measurements without requiring programming expertise. New neurofeedback protocols can be easily created, saved, and retrieved. We provide an example code for real-time data preprocessing and visual feedback; however, users can customize or expand it with appropriate programming skills.

**Conclusions:**

NINFA enables real-time recording, analysis, and feedback of brain signals. We were able to demonstrate the stability and reliability of the computational performance of preprocessing and analysis methods in the current version. NINFA is intended as an application that can, should, and may evolve with the help of contributions from the community.

## 1 Introduction

Neurofeedback (NF) has emerged as a promising tool in neuroscience for facilitating self-regulation of neural activity.^1^^–^^4^ By providing real-time feedback on brain activity, NF allows individuals to modulate their own neural processes. NF training protocols find application across a diverse spectrum of research domains, spanning from augmenting cognitive functions in healthy individuals to ameliorating behaviors associated with neurocognitive disorders.^5^ The efficacy of NF training is underscored by its ability to induce specific neural changes within trained brain circuits, leading to behavioral changes.^4^ The effects of NF training have been demonstrated to persist beyond the training period, ranging from hours to months following the completion of training.^6^ Furthermore, these effects have been found to correspond with alterations in the structure of both gray and white matter within the brain.^7^ Recent research has shed light on the neural circuitry involved in brain self-regulation, implicating regions such as the thalamus and dorsolateral prefrontal, posterior parietal, and occipital cortices in NF control, and the dorsal and ventral striatum, anterior cingulate cortex, and anterior insula in NF reward processing.^8^

The NF methodological pursuit commences with the measurement of selected parameters of brain function, facilitated by an arsenal of sophisticated techniques.^5^ Electrophysiological methodologies, including electroencephalography (EEG), magnetoencephalography (MEG), and invasive electrocorticography, afford profound insights into the intricate dynamics of neural signaling.^4^ Concurrently, hemodynamic imaging modalities such as functional magnetic resonance imaging (fMRI) and functional near-infrared spectroscopy (fNIRS) offer elucidation into the dynamic interplay between neural activity and hemodynamic responses.^9^^,^^10^ These multifaceted methodologies serve as the cornerstone for deciphering the enigmatic workings of the brain and harnessing its potential for self-regulation through NF.

Among the array of neuroimaging modalities available, fNIRS emerges as a promising tool in the field of NF.^10^ By emitting near-infrared light (650–950 nm) into the cortex, fNIRS measures brain activation by quantifying concentration changes of oxygenated and deoxygenated hemoglobin in response to neuronal activity.^11^^–^^13^ fNIRS offers a spatial and temporal resolution between fMRI and EEG. With a spatial resolution of approximately 2–3 cm, fNIRS surpasses EEG. fNIRS has a lower spatial resolution and signal-to-noise ratio than fMRI; however, it may have a higher temporal resolution than fMRI due to its higher sampling rate.^3^ Its low spatial resolution compared with fMRI limits the ability to precisely target specific small brain areas. Moreover, the technique does not provide individualized anatomical images, which can make precise targeting of brain regions of interest more difficult. The limited penetration depth of infrared light, which only reaches the most superficial cortical layers, is also an obstacle to using fNIRS for NF on deep brain areas, such as the basal ganglia or hippocampus. However, the ability to measure both oxyhemoglobin and deoxyhemoglobin ensures more personalized feedback, adaptable to the patient’s individual needs.

However, its distinct advantages, including cost-effectiveness, accessibility, and reduced susceptibility to motion artifacts, pose fNIRS as a versatile tool with significant translational research and clinical application potential in the NF field.^1^^–^^3^ Unlike fMRI, which poses logistical challenges due to its massive equipment and stringent operational requirements, fNIRS offers portability, ease of use, and silent operation,^10^ making it applicable for use in diverse settings, including clinical environments^14^ and naturalistic scenarios.^15^ Moreover, its tolerance to motion artifacts surpasses that of EEG and fMRI, enabling investigations in populations prone to movement disturbances, such as neurological patients^16^ and infants.^17^ Furthermore, the speed of preparation and the absence of the need for gels or other contact materials make it an easier instrument to use, even by nonspecialized personnel or even by the patient himself, increasing the frequency of sessions and thus improving NF results.

Real-time processing of fNIRS data, like all real-time data processing, must deal with certain challenges associated with the method.^2^ fNIRS records neuronal activity indirectly via changes in blood flow and blood (de)oxygenation in the cortical tissue. Neurovascular coupling is the complex relationship between neuronal activity and cerebral blood flow. An increase in oxygenated hemoglobin (HbO) with a simultaneous decrease in deoxygenated hemoglobin (HbR) indicates increased oxygenation of the blood and thus activation of the brain.^11^ This so-called hemodynamic response takes 4-6 s to reach its maximum amplitude leading to a relatively poor temporal resolution, which needs to be taken into account for real-time fNIRS-based NF application.^18^ Another challenge with fNIRS is the presence of physiological signals that do not reflect brain activity in the brain such as periodic components corresponding to cardiac, respiratory, and other blood flow regulation dynamics or slow drifts.^19^ Slower oscillations are the most challenging periodic components to eliminate in real time. Indeed, the lower the frequency of the component, the fewer samples there are within a certain time window, thus increasing the challenge of removing that component.

Another critical factor is the variability of the signal among individuals due to physical characteristics such as skull thickness, skin pigmentation, and hair density,^20^ which may influence the quality of the measurement. This implies that NF based on fNIRS may not be equally effective for all patients, limiting its universality. In addition, brain coverage is often limited by the number of available optodes, and increasing the number of optodes to improve spatial resolution may lead to greater patient discomfort, compromising tolerability. From a technical point of view, fNIRS is sensitive to physiological noise, such as variations in blood pressure and heart rate, which can introduce noise into the signal and reduce the reliability of NF. The low temporal resolution, due to the delay of the hemodynamic response, can slow down the learning and effectiveness of NF, making it less immediate than techniques such as EEG. Finally, although generally well-tolerated, prolonged use of the fNIRS band or helmet may be uncomfortable for some users, impacting their adherence to NF sessions.

Despite these limitations, many of them can be mitigated with advanced technological solutions, such as the integration with other techniques (EEG and fMRI), the development of high-density systems (HD-DOT), and the application of algorithms for real-time motion and systemic artifact correction. Overall, fNIRS appears to be a promising technology for NF, especially in clinical settings and for sensitive patients, with room for improvement that could further extend its effectiveness and applicability.

Real-time analyses are the basis for NF as they enable immediate feedback on certain parameters of brain activation.^5^ Constantly incoming data must be processed continuously within a very short period of time using sufficiently fast calculations.^21^ In real-time processing, the use of effective and robust real-time preprocessing techniques is therefore essential to improve the accuracy of the measurements and the feedback provided to the user. The resulting increased reliability of the NF signal could, on the one hand, improve the outcome of the intervention and, on the other hand, improve the controllability of the feedback signal by the user and thereby increase their compliance. fNIRS NF is still a relatively young field, and previous NF studies were mostly conducted without real-time artifact corrections.^3^ This could be due, among other things, to a lack of appropriate recommendations and validations of offline corrections for real-time processing. Recently, there have been initial reviews that have carefully listed the challenges and possible solutions.^2^^,^^22^ This provides a good starting point to address the lack of standardized analysis pipelines, and then, open-source options pave the way for more uniform approaches and community-based continuous improvements. Although real-time analysis software for fNIRS already exists,^21^ it is not freely accessible to the broader scientific community, limiting opportunities for widespread use and collaboration in the field of NF. Considering the potential efficacy of employing fNIRS within the realm of NF, our objective is to provide an open-access tool designed for the execution of real-time NF experiments. The application is MATLAB-based, which means that it is openly accessible, but a MATLAB license is required to use it, which still limits the full open-access concept. However, many have full access to MATLAB through their research institutions. One of the main advantages of MATLAB is the reliability of the results, thanks to well-documented and constantly updated toolboxes covering a wide range of scientific fields. Furthermore, the use of these predefined libraries enables accurate results without the need to develop code entirely from the beginning, making MATLAB a powerful and efficient tool for data analysis. A further strength is the dedicated technical support: users can contact the MATLAB team and receive direct assistance for specific problems.

## 2 Methods

The NINFA 1.2.0 application is written in MATLAB R2020a (MathWorks, Natick, Massachusetts, United States). MATLAB is a high-level language and interactive environment for numerical computation, visualization, and programming. We used the MATLAB App Designer environment to create the NINFA 1.2.0 application, which incorporates the user interface and integrates the developed algorithms. The MATLAB App Designer allows users to design apps with an individually organized graphical user interface (GUI). The NINFA 1.2.0 application is the starting point to access developed algorithms for real-time acquisition and to organize study protocols. We have developed and tested NINFA 1.2.0 in combination with the NIRx NIRSport2 system (NIRx Medical Technologies, LLC, Berlin, Germany) and the Aurora v2021.9 fNIRS acquisition software for NIRSport2 connected via lab streaming layer (LSL).^23^ The computer involved Intel^®^ Xeon^®^ Silver 4208 CPU @ 2.10 GHz, and 32 GB RAM.

### 2.1 Application Components

To use NINFA 1.2.0 for a real-time fNIRS-NF, in addition to the acquisition hardware, the user needs a computer on which the acquisition software and NINFA 1.2.0 are installed (or two computers that can be connected together) and an extended screen connected to this computer to display visual feedback to the participant. The NINFA 1.2.0 application can be downloaded from GitHub (https://github.com/PsychoOI/NINFA) in the form of a zip file. The folder contains a MATLAB script, *main.m*, with which the GUI can be opened, as well as six further subfolders:

• The **components** folder contains the *device.m* function that selects the appropriate device type (e.g., EEG or NIRS) and loads the corresponding JSON file (see below); the *lsl.m* function that allows the connection via LSL with other devices; the *protocol.m* function that checks the requirements for the selected protocol, including the type and number of NF channels; and the *session.m* function that stores all information about a session (start, stop, and update a running session).
• The **devices** folder contains settings of different devices in JSON files (i.e., the structure of the data stream).
• The **liblsl-Matlab** folder contains the main library used for handling data streams among devices. liblsl-Matlab is an open-source library designed for integrating real-time data streams, and it is available on GitHub.^24^
• The **protocols** folder contains the function to execute both the preprocessing and the NF computation.
• The **sessions** folder contains the data output of each recording.
• The **settings** folder contains the settings of a specific real-time protocol (see Sec. 2.2.2).
• The **ui** folder contains the *app.mlapp* file that defines the main structure of the application; the *selectedchannels.m* file that allows the user to easily select channels and save their choices; and the *feedback.m* file that is responsible for generating feedback.

To ensure a basic understanding of how to use the NINFA 1.2.0 application, we will further explain the key components in Sec. 2.2 (see also short tutorial video).^53^

### 2.2 GUI

**Fig. 1:**
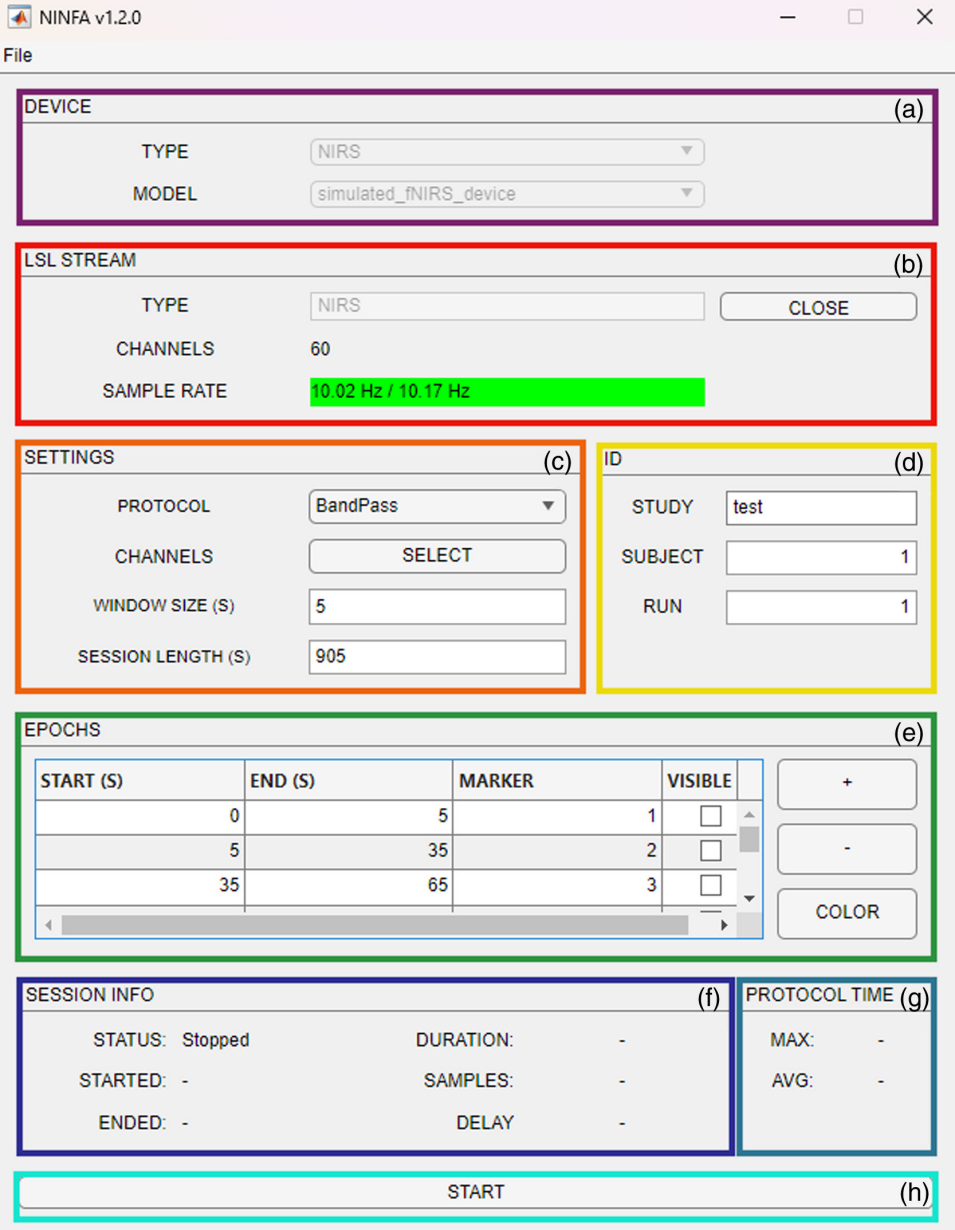
GUI of NINFA 1.2.0. (a) The DEVICE section contains information on the type (TYPE) and name (MODEL) of the device connected to the application. (b) The LSL STREAM section includes information on the type (TYPE) of the device, the number of CHANNELS available as well as the SAMPLE RATE. (c) The SETTINGS window includes configurations to set up the real-time analysis (PROTOCOL), the CHANNELS selected, the WINDOW SIZE (in seconds) during which real-time-analysis is performed, as well as the SESSION LENGTH in seconds. (d) The information entered in the ID text boxes is used for naming the generated data file. (e) The EPOCHS section contains information on the events or conditions of the experiment, such as the sequence, duration, marker number and number of markers, as well as the visual appearance of the participant (e.g., the visibility of the thermometer and the background color). (f) The SESSION INFO displays the status information for the recording. (g) The PROTOCOL TIME section informs the user whether real-time analysis of the data is causing a delay or whether it is computationally efficient enough. (h) The START button allows to start the acquisition.

Figure 1 shows the GUI of the NINFA 1.2.0 application. Eight different panels are present in the main application window, which serves as a starting point from which relevant functions and saved protocols can be accessed, new protocols can be created and saved, and ongoing recordings can be monitored by the examiner. In Sec. 2.2, we will go through the individual points in more detail and provide a step-by-step guide to the procedure for using NINFA 1.2.0 for an fNIRS-NF. Moreover, the functions of the GUI are described in detail in this section.

#### 2.2.1 Device

NINFA 1.2.0 is designed to offer the greatest possible flexibility when combining different data acquisition software and hardware. This makes it possible to create a configuration that is customized to the respective measurement “TYPE” (currently EEG and NIRS are available), device, and the channel configuration used. LSL is a protocol that enables the exchange of experimental data in real time, and it can be easily synchronized between NINFA 1.2.0 and the acquisition device. To make this possible, a new configuration must be created and stored in a JSON file in the **devices** folder that contains information about the exact arrangement of the information that is transmitted via the LSL connection. This JSON file must contain information about the “name” of the device (e.g., “NIRx NIRSport2 (26CH)”), the “type” of data (e.g., “NIRS” and “EEG”), and which columns in the LSL stream represent, e.g., for NIRx NIRSport2, the frame counter, raw voltage data for the respective wavelengths, oxyhemoglobin data and deoxyhemoglobin data, and their respective units if available (e.g., “V” and “μmol/L”). The **devices** folder in the GitHub repository contains an example of a JSON file for use with an fNIRS 20-channel configuration and two wavelengths with the NIRx NIRSport2 (nirs_nirx_nirsport2.json) as well as for 26 channels (nirs_nirx_nirsport2_26.json). There is also a generic file for fNIRS and one for EEG, which can be used as a template for creating a new JSON file. In the NINFA 1.2.0 main application window, the name in the textbox near “MODEL” corresponds to the device (i.e., the JSON file) you selected from the **devices** folder. After creating a new configuration, NINFA 1.2.0 must be restarted.

#### 2.2.2 LSL stream

The LSL is an open-source framework designed to facilitate the collection, synchronization, and real-time transmission of time series data in research settings. It is commonly used with data to integrate from different resources, such as neural, physiological, and behavioral sensors.^23^ Before starting an experiment, the devices (the computer where the NINFA 1.2.0 application is installed, the computer where the acquisition software is installed, and the fNIRS hardware) must be connected to a common network. Before starting a real-time acquisition, the LSL connection needs to be established, which is executed automatically when *main.m* is started. After this, the connection among the devices should be checked. As soon as the connection has been successfully established, the NINFA 1.2.0 application can be opened. To do this, the current path in MATLAB must be set to the path of the NINFA 1.2.0 application. After opening *main.m*, the user clicks the Run button in the MATLAB menu bar to open the GUI shown in Figure 1. Then, in NINFA 1.2.0, the user clicks the “OPEN” button in the GUI. The name “NIRS” in the textbox near “TYPE” corresponds to the LSL stream name. After clicking the “OPEN” button, if the connection is successful, the button label changes to “CLOSE,” and the number of available channels and the fNIRS sampling rate value are displayed next to the “CHANNELS” and “SAMPLE RATE” text labels, respectively. Otherwise, if after clicking the “OPEN” button, the connection is not successful, a warning box will appear to inform the user that the connection failed. The “CLOSE” button must be clicked when the user wants to stop the connection.

#### 2.2.3 Settings

**Fig. 2:**
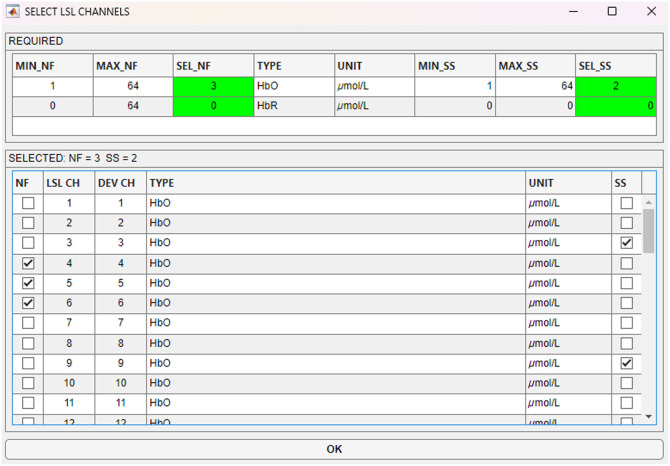
GUI of NINFA 1.2.0 for channel selection. REQUIRED: Protocols have minimum channel requirements that must be selected to continue (which is then indicated by a green label).

This section of the application contains various information about the NF protocol and the calculation of the feedback signal. Once the connection is established, the user can click on the “Select” button next to “CHANNELS” to select the channels to be used for the calculation of the NF signal from a dropdown list (see Fig. 2). The example protocol *MovAvg_SS.m* specifies that the user must select at least one channel for NF and one for short-separation regression, with HbO values in the unit micromole per liter. This minimum requirement, which can of course also be defined differently (e.g., at least eight channels or at least one HbO and one HbR channel), reduces the susceptibility to errors when using the application. In addition to the NF channels, the settings panel also contains:

• “PROTOCOL,” which allows loading the file for the real-time preprocessing, analysis, and NF computation. The dropdown list for the protocol choice shows MATLAB functions that are saved in the **protocols** folder. These functions can be modified by the user to fit their requests. Here we propose an exemplary preprocessing pipeline *MovAvg_SS.m* for a real-time fNIRS NF application (see Sec. 2.3.2). Changing or adding a preprocessing pipeline for real-time NF in NINFA 1.2.0 requires significant technical and programming knowledge.
• “WINDOW SIZE (S),” to set the number of seconds for each time window for real-time preprocessing. The required time window can vary not only depending on the sample frequency but also depending on the selected preprocessing method and the data points required for its calculation. The window size can be easily changed by the user.
• “SESSION LENGTH (S),” to set the duration of the NF run (or a complete session of several runs). The duration results from the total duration of the experimental protocol defined in the Epochs panel (see Sec. 2.2.5 for more details). The session length can be easily changed by the user.

#### 2.2.4 ID

The ID panel allows the user to save the data correctly. The “STUDY,” “SUBJECT,” and “RUN” fields are used to decide the final name of the output file (i.e., *Study_Subject_Run.mat*). The data is saved in the **sessions** folder.

#### 2.2.5 Epochs

The experimental protocol is defined in this panel. Each row represents an event (e.g., regulation, rest, or pause) and is identified by the start time “Start (s),” the end time “End (s),” the number of the marker type “Marker,” as well as the “Visibility” of the visual feedback on-screen during an event and the color of the display background. The marker type number allows the event to be classified (i.e., regulation, rest or pause) and is stored both in the application and in the.snirf data in Aurora. The markers are sent to the acquisition software via LSL. By checking or unchecking the checkbox in the “visible” column in the corresponding event row, one can determine whether the thermometer is displayed in this event or not. To add a row, i.e., an event, click the “+” button, and to remove a row, click the “-” button. The “Color” button can be used to change the background color in a selected row. The color setting can be useful to give information to the participants during the experiment (e.g., as a prompt for the conditions of rest versus regulation). This procedure must be carried out manually to create a new experimental protocol, which can then be saved (see Sec. 2.2.6). This allows the user to automatically load the configuration after the first saving.

#### 2.2.6 File

After building the experimental protocol in the NINFA 1.2.0 application (Settings, ID, and Epochs), it is possible to save the preferences by clicking on “File—Save.” If the experimental protocol was already saved and you want to load it before starting a real-time acquisition, it is possible by clicking on “File—Load” and selecting the configuration.

#### 2.2.7 Session info

**Fig. 3:**
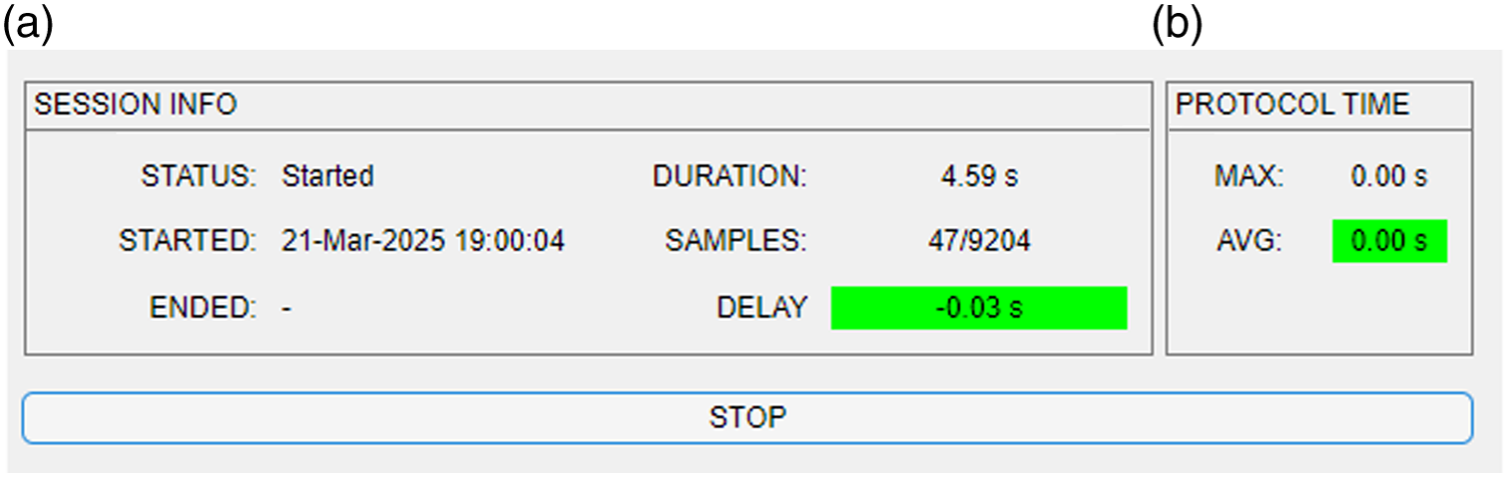
(a) SESSION INFO section of the GUI of NINFA 1.2.0 during an ongoing assessment. (b) PROTOCOL TIME section of GUI of NINFA 1.2.0 during an ongoing assessment.

This section serves to monitor the recording and is updated as soon as an acquisition has started until the end of the recording. It returns the number of the instantaneous frame out of the total frames (SAMPLES). The delay component (DELAY) is very important for monitoring a running real-time session. This parameter indicates whether the preprocessing is too computationally intensive. To obtain the value of delay, the expected number of samples recorded during the session, from the beginning to the current time, is first calculated. Then, the lost frames are calculated by subtracting the acquired frames from the expected number of samples. Finally, the delay is calculated as the difference between the expected time and the actual time (times were obtained by dividing the number of samples by the sampling frequency). If the computation time increases too much, the procedure is no longer in real-time resulting in an increasing delay. We assumed that if the difference was greater than 1 s, the delay was considered unacceptable. To better highlight problems with the delay component, the label is colored: a green label corresponds to a negligible delay [see Fig. 3(a)], whereas a red label corresponds to a markable time delay in the NF signal calculation. If this is the case, one option would be to use a more powerful hardware or the preprocessing needs to be less computing-intensive. A third option would be not to perform the calculations for every window, but only for every second or third window, etc. A fourth option would be to reduce the sample rate used on the device. As soon as the acquisition begins, the STATUS changes from Stopped (see Fig. 1) to Started [see Fig. 3(a)]. The date and time of the start (STARTED) and the end (ENDED) of the acquisition are shown in this panel. Finally, in this panel, the time in seconds elapsed since the beginning is also reported (DURATION).

#### 2.2.8 Protocol time

This section [see Fig. 3(b)] supervises the performance of real-time preprocessing during an ongoing assessment. The max value corresponds to the maximum execution time among the tracked processing, whereas the avg value corresponds to the average execution time of the tracked processing. The avg value monitors the performance of real-time computation to ensure it does not exceed the predefined time limit of 1  s/sample rate during protocol execution resulting in delays (see above). If the limit time is exceeded, then the avg color label changes from green to red.

#### 2.2.9 Start

After clicking the “START” button, the acquisition will begin, and the label turns to “STOP.” The acquisition will finish after reaching the experiment DURATION or by clicking on the “STOP” button. Then, the “START” label will appear again.

#### 2.2.10 NF display

**Fig. 4:**
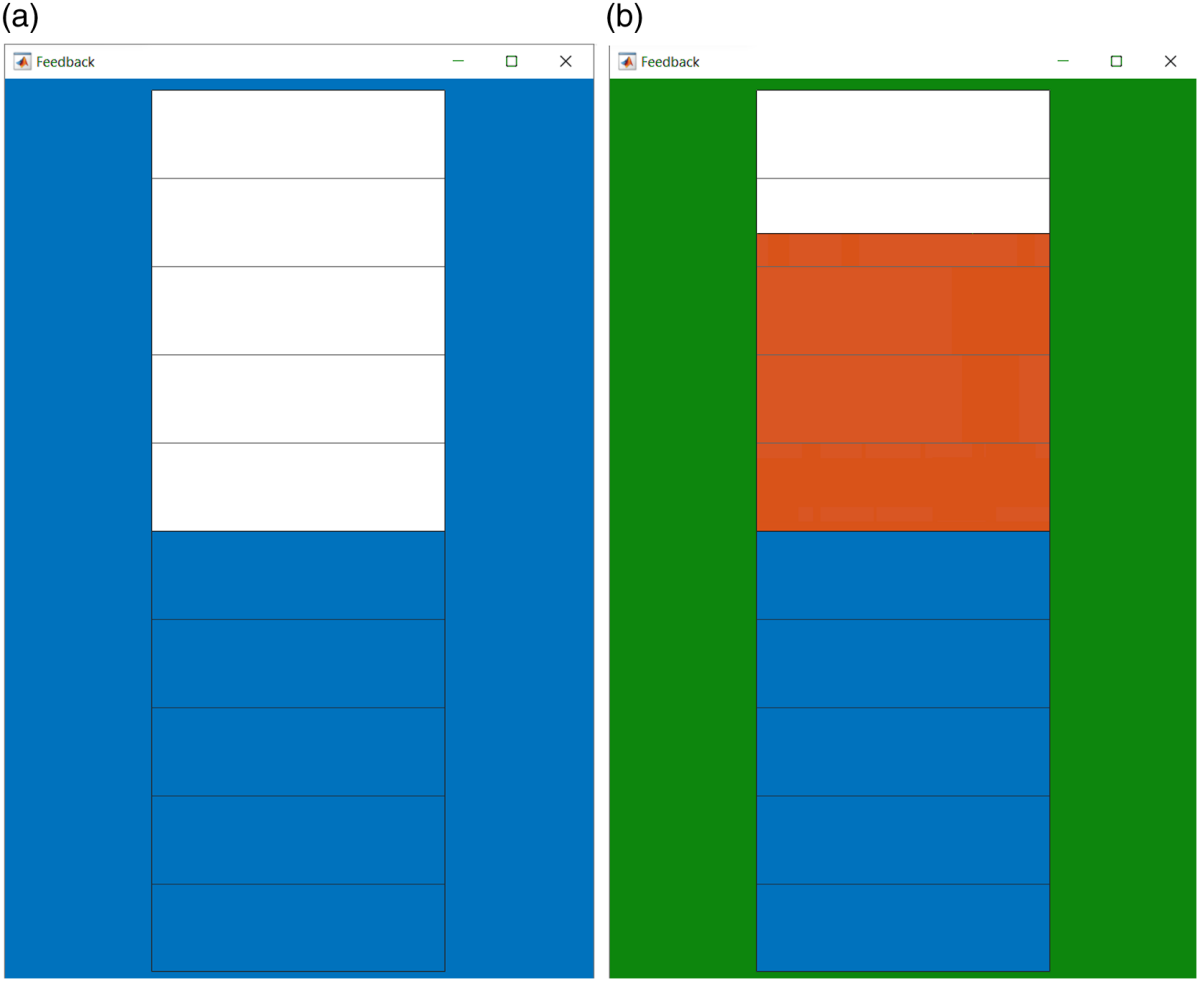
NINFA 1.2.0 NF window shows the currently implemented visual feedback in the form of a thermometer during rest (a) and regulation (b). The background of the thermometer indicates the current condition to the subject: rest (blue) or regulation (green).

Another key component in NF involves the feedback of the preprocessed signal in real time. Visual feedback stands out as the most prevalent in existing literature,^25^ yet alternative modalities such as auditory or haptic feedback are also feasible. In the current version, NINFA 1.2.0 only offers a visual representation of the feedback signal in the form of a thermometer with different background colors (see Fig. 4). In the following section, we describe an exemplary method for calculating the feedback to be returned to the participant, however, with good programming skills, it is possible to change the calculation method. The selection of whether or not feedback should be displayed for each event, as well as the color of the thermometer background, can be easily changed via the GUI (see Sec. 2.2.5).

### 2.3 Example of Experimental Procedures

**Fig. 5:**
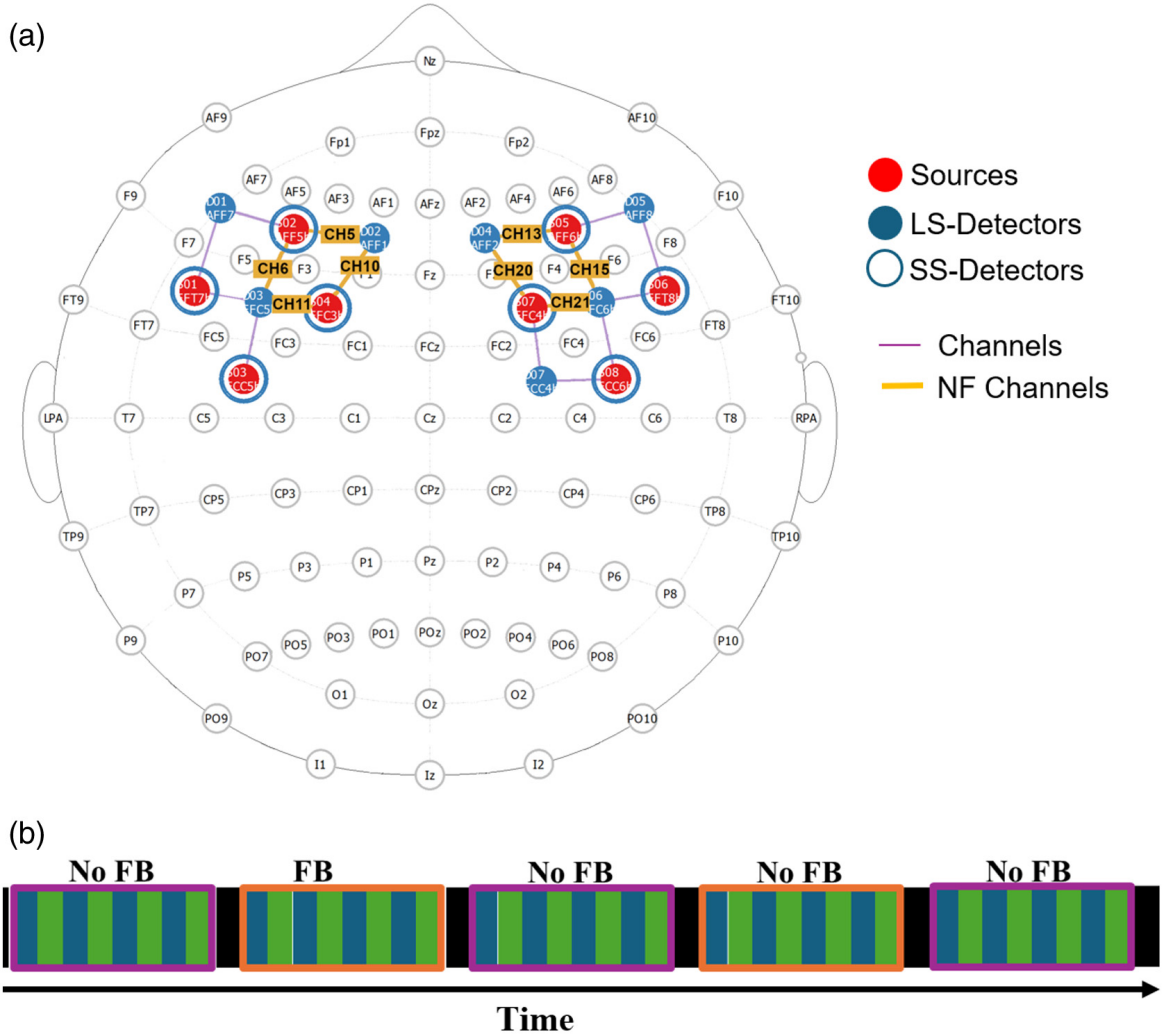
(a) Optode montage with yellow highlighted NF channels used for the experiment. (b) The session is divided into five main runs. Purple blocks represent NoFB conditions, and each consists of five 30-s regulation phases (green bars), each separated by a 30-s rest phase (blue bars). Orange blocks indicate feedback conditions, and each consists of six 30-s regulation phase (green bars), each separated by a 30-s rest phase (blue bars). Black segments represent breaks, shown as black screens with no instructions, and in which participants can move, talk, drink, and relax among runs. CH, channel; D, detector; LS, long separation; NF, neurofeedback; S, source; and SS, short separation.

To help the user understand the NINFA 1.2.0 application, we report an example that includes all the steps for developing an fNIRS NF setup and acquisition (see Fig. 5). The example protocol as well as the underlying functions to preprocess the fNIRS data in real time and detailed instructions are available on GitHub (https://github.com/PsychoOI/NINFA).

#### 2.3.1 Experimental protocol

Initially, we tested the code using some theoretical simulations, and after some adjustments, we tested the code in a finger-tapping task, motor imagery, and NF of prefrontal hemodynamic response with the help of several lab members. We obtained informed consent to the study. The newly established fNIRS-based NF training protocol was approved by the ethics committee at the University Tübingen and University Hospital Tübingen for studies in healthy participants (548/2023BO1); the data reported here were derived from the first pilot trainings conducted in healthy participants (i.e., lab members). The first sanity check that followed the simulations was a test using motor execution. This decision was based on the intention to induce as strong a signal change as possible to initially effectively test and adapt the performance of the code. In the next step, we tested the code using motor imagery because it is a fairly simple and easily accessible type of NF for the participants. Only in the last step did we test the code with the prefrontal hemodynamic response because here the hemodynamic response is less pronounced, less clearly demarcated between rest and task, and because the regulation of the prefrontal hemodynamic response is more difficult (see the Supplementary Material for some notes on our experience). After further adjustments to the code following the first practical tests, we measured five members of our research group in an NF of prefrontal activity to recheck the plausibility of our example protocol within the NINFA 1.2.0 application now published on GitHub. fNIRS NF of the prefrontal cortex is currently the most common application of fNIRS NF.^3^ fNIRS data were acquired with a continuous-wave, portable, multichannel fNIRS system (NIRSport 2, NIRx Medical Technologies, Berlin, Germany) with the acquisition software Aurora fNIRS v2021.9 (NIRx Medical Technologies, Berlin, Germany). To target the dorsolateral prefrontal cortex reliably, the placement of the optodes was first checked using fOLD v2.2.1.^26^ The montages, i.e., the location of sources, detectors, and their connections (i.e., the channels), were then created in the software NIRSite v2.0 (NIRx Medical Technologies, LLC, Berlin, Germany) using the standard 10–10 system. The optode positions can also be displayed in the MNI space, which also helps to set the positions as precisely as possible. This led to the definition of eight NF channels (5, 6, 10, 11, 13, 15, 20, and 21) corresponding to the dorsolateral prefrontal cortex. The montage consisted of eight LED illumination sources and eight detectors. The array was composed of 18 standard channels (3 cm) and 8 short separation (SS) channels (8 mm) [see Fig. 5(a)]. The sampling frequency was set at 10.17 Hz. During acquisition, the participant sat on a chair in a quiet and dimly lit room.

The session procedure is depicted in Fig. 5(b). As we had defined a sliding window size of 5 s, a session began with a 5-s phase (black screen), required to wait with any calculations until the first window was fully available. This was followed by five 30-s regulation phases (green screen), and separated by 30-s rest each (blue screen). In the regulation phases (green screen), the participants were instructed to use strategies that activate the prefrontal cortex and to let their minds wander as soon as the screen turned blue (rest phase). We tested the NF of the prefrontal hemodynamic response with a paradigm that we plan to actually use in a study in the future. In this paradigm, the fNIRS NF will be combined with simultaneous transcranial direct current stimulation (tDCS). Because the tDCS can only run continuously for a maximum of 30 min and the participants need a (in this case precisely timed) break (black screen) to drink, move, talk, and relax, we shortened the two transfer runs by one trial each. As we tested members of our working group who, due to their expertise, have a fairly precise idea of possible strategies for activating the prefrontal cortex, we did not give any further instructions on this. Their strategies included to-do lists,^27^ math problems,^28^ or verbal fluency tasks.^29^

The 30-s regulation phases and the 30-s rest phases in between without feedback are common procedures in fNIRS NF studies.^14^^,^^30^^–^^32^ The 30-s rest phase without feedback is necessary to allow the hemodynamic response to return to its baseline level so that the baseline for the upcoming trial can be calculated without mixing task-related activity from the previous regulation phase. During the rest phase, participants are supposed to relax and therefore receive no feedback (NoFB). However, there are also some studies applying hemodynamic NF in which feedback was continued during the rest phase.^33^

The participants received NoFB on their brain activity during the first run. Then, the five trials were followed by a 30-s break (black screen), during which the participants could relax. The following three runs consisted of six 30-s regulation phases each, separated by 30-s breaks. In these runs, participants received feedback on their hemodynamic response to prefrontal brain activation in the form of a thermometer that was filled with red bars when strategies successfully enhanced the hemodynamic response to prefrontal activation compared with baseline or fell when strategies resulted in a decrease of prefrontal cortex activation. The screen colors were identical to before, and a blue screen background indicated the rest phase (the thermometer was displayed during this phase but did not move and was filled with blue up to the midline), whereas a green screen indicated a regulation phase. The last run again consisted of five regulation trials in which no thermometer was displayed, but only a change in the screen colors indicated the task phase (regulation versus rest).

#### 2.3.2 Setting up a new NF protocol in NINFA 1.2.0

With NINFA 1.2.0, we propose a novel open-access approach for real-time preprocessing and NF signal calculation. In our setup, the LSL protocol enables communication between the NINFA 1.2.0-MATLAB scripts and the NIRx Aurora software. Aurora connects with the fNIRS hardware, acquires the data, and converts the raw light intensity to HbO and HbR concentration using the modified Beer–Lambert law. The conversion parameters are invariant in Aurora (wavelength 760 nm absorption coefficients for HbR 3.57 and for HbO 1.35; differential pathlength factor: 6.30; wavelength 850 nm absorption coefficients for HbR 1.59 and for HbO 2.44; differential pathlength factor: 5.23). However, to perform the modified Beer–Lambert law, Aurora needed 120 s of baseline at the very beginning. So we waited to start the session until this time was reached. It is important that during the baseline the participant remains as still as possible to avoid motion artifacts in the fNIRS signal. Then, Aurora transmits the fNIRS data using the LSL protocol. NINFA 1.2.0 receives the fNIRS data, preprocesses the data, calculates the feedback signal, updates the visual display in the GUI for the user to monitor the recording, and updates the visual feedback for the participant.

To register the desired NF protocol in NINFA 1.2.0, we first defined the detailed experimental setup parameters in the fields provided. In the DEVICE section, we selected the previously created “nirs_nirx_nirsport26.json,” in which the information about the structure of the LSL stream is stored. The Aurora LSL Stream for NIRx nirsport2 consists of a vector with dimension (N*4)+1, where N corresponds to the number of channels. The first position counts the number of frames during the acquisition, and then, in the array, there are N raw light intensity data for the first wavelength, N raw light intensity data for the second wavelength, N oxyhemoglobin (HbO) concentration changes values, and N deoxyhemoglobin (HbR) concentration changes values. You can create a new device according to the configuration of your data stream by simply copying one of the existing files and adjusting the information in the file to the number of channels of your montage and the number and/or sequence wavelengths and saving it under a meaningful name for that specific montage. After creating a new device, you must restart NINFA 1.2.0.

In the SETTINGS section, we defined eight NF channels (5, 6, 10, 11, 13, 15, 20, and 21) corresponding to the dorsolateral prefrontal cortex [see Fig. 5(a)], a window size of 5 s, and a session length of 1800 s. In the ID section, for study, we entered “test”, and we numbered each subject starting with “1” as we recorded only a single session per participant, we entered “1” in the run text field. Then, we selected our protocol using the dropdown list. To define the sequence of events, we added 61 new lines in the EPOCH field: The first event (marker ID 1; black background color) of 5 s duration served to collect a sufficiently large number of samples for the following computation. Then, we entered the start and end time for each of the rest (marker ID 2; blue background color) and regulation phases (marker ID 3; green background color) as well as break among runs (marker ID 1; black background color). Each row started with the “end” frame of the previous row and ended 30 s later. There were 28 task–rest repetitions and four breaks among runs.

#### 2.3.3 Exemplary pipeline for real-time preprocessing and feedback calculation in NINFA 1.2.0

Compared with offline analyses, real-time fNIRS signal correction encounters several challenges. Indeed, although offline acquisitions utilize the entire signal, real-time acquisitions employ short sliding temporal windows (∼1−5  s) leading to a smaller number of samples available for preprocessing and analyses. Therefore, computationally intensive functions (e.g., Wavelet filtering) cannot be utilized as they cause data acquisition delays, and filtering low frequencies becomes challenging due to the short duration of the sliding windows. Various approaches have been employed in the literature to deal with fNIRS data using different equipment,^19^ whereas limited information exists regarding the online processing of fNIRS data in real-time.^2^^,^^22^^,^^34^^,^^35^ In our exemplary approach, the real-time preprocessing steps were implemented using oxyhemoglobin concentration changes (μM) and a 5-s sliding window approach. Although HbR is less susceptible to artifacts, the amplitudes are also lower than those of HbO, which in turn is more susceptible to artifacts.^36^ In the majority of NF studies, HbO has been chosen as the feedback parameter (most probably) due to the larger amplitudes, although there has often been no real-time correction.^3^ In addition, we applied a 5-s sliding window to smooth the signal and improve the reliability of the feedback by reducing short-term fluctuations. Participants are generally informed that their response is not immediate as the hemodynamic nature of fNIRS inherently introduces a delay. Applying a 5-s moving average may slightly extend this delay, but this approach is widely used in the literature.^3^ A 5-s window, in particular, represents a good balance between keeping the delay manageable and ensuring feasible signal processing.

With each new sample, the sliding window slides to the next sample, and the oldest sample is discarded, thereby maintaining a constant window size. Each time series stream (5 s length) was subjected to real-time preprocessing employing a short separation channel regression and a moving averaging filter. The real-time preprocessing algorithm *MovAvg_SS.m* provided as an example with NINFA 1.2.0 contains 4 functions: 1) requires() checks if the requirements for this algorithm are met (e.g., must be NIRS data, at least one HbO channel is required, min/max supported sliding window sizes that one should configure in the “SETTINGS” when using this protocol). The *MovAvg_SS.m* function is only shown if at least one HbO channel is present. 2) init() initializes the preprocessing steps at the beginning of each session. 3) process() is called in each sliding window. 4) finish() can be used to do any kind of post-processing on the whole session data (e.g., create a graph at the end of the run or the session). The four functions mentioned must be available in all protocols - with possibly different implementations within the protocols, of course.

For the continuous feedback within the NINFA 1.2.0 application, we have implemented the picture of a thermometer whose temperature, i.e., filled bars, reflect HbO amplitude increases (red bars) or HbO amplitude decreases (blue bars) of the fNIRS signal in the target area, relative to the baseline phase. The average of the activation in the baseline is depicted as a half-filled thermometer. Values above this, i.e., more filled bars, indicate an increased activation, whereas values below this line, i.e., less filled bars, indicate decreased activation, compared with the baseline (see Fig. 4). We have opted for a half-filled thermometer to depict the baseline level so that we can also feedback HbO compared with the baseline (which can also decrease compared with baseline). There are also some examples of this in the literature.^37^^,^^38^

To visualize the mean hemodynamic response of the NF channels during the regulation phase relative to the baseline, the HbO value must be converted into a corresponding filling level of the thermometer. We used a min–max normalization that adjusts the HbO values to fit within the thermometer range, which we defined as 0 to 1. Thus, the thermometer spans from 0 to 1 and is divided into increments of 0.1 units. The average of the activation in the rest phase is depicted as a half-filled thermometer, corresponding to a value of 0.5 in the thermometer range. Feedback values <0.5 in the thermometer range, i.e., a decrease relative to the baseline, are visualized in blue. Values >0.5 in the thermometer range, i.e., an increase relative to the baseline, are visualized in red.

**Fig. 6:**
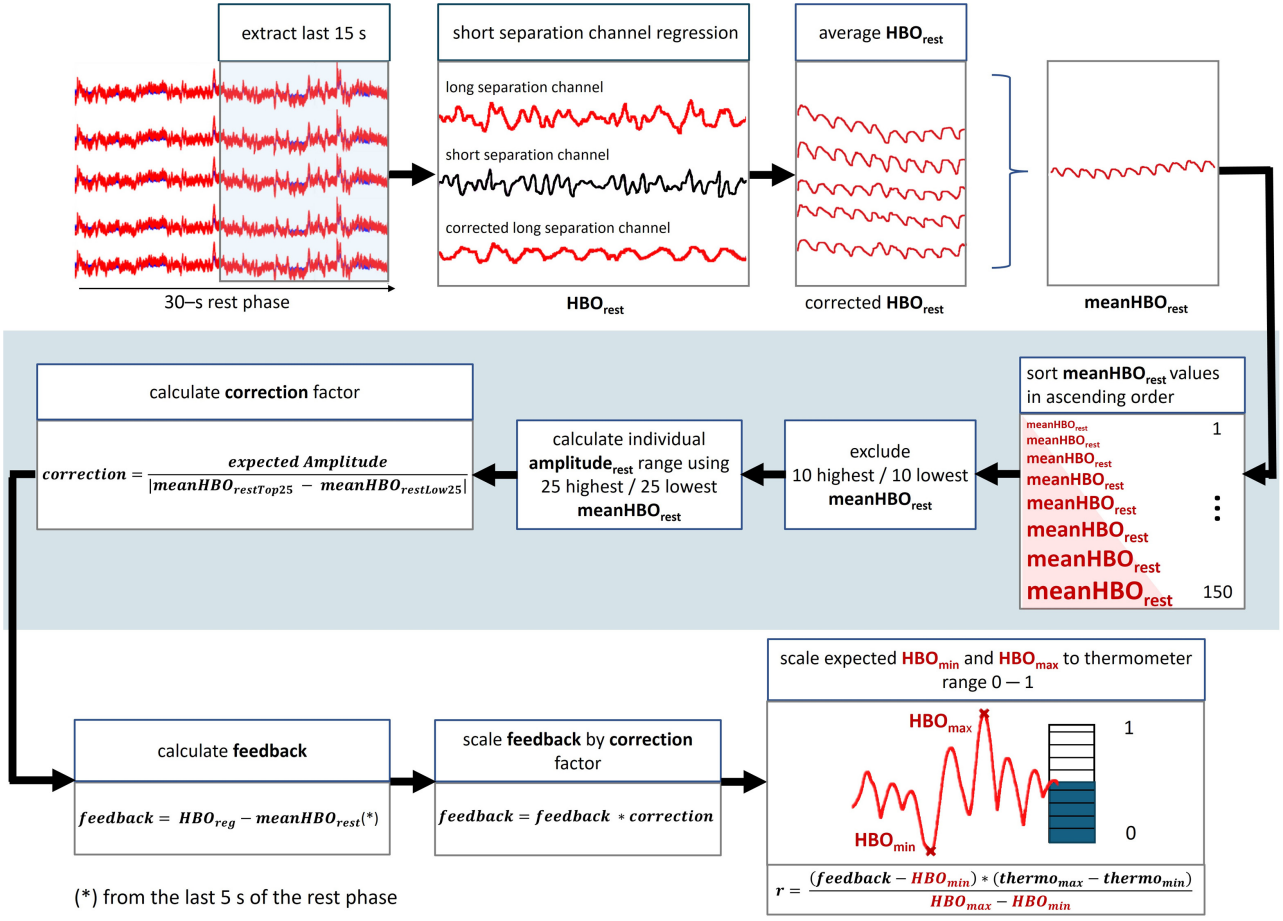
Preprocessing and generation of feedback, as implemented in our *MovAvg_SS.m* protocol example. Please note that the figures are for illustrative purposes only and do not represent real data from the pilot measurements. They are therefore not intended for analytical interpretation.

To convert the HbO amplitudes into a corresponding fill level of the thermometer, several steps are performed, as shown in Fig. 6. First, the HbO values from the last 15 s of the first rest phase are extracted for each NF channel, referred to as HBOrest. Each HBOrest is then filtered, and an average channel (meanHBOrest) is created by calculating the average across NF channels. These values are sorted in ascending order (∼150 samples in this example). To reduce the influence of potential outlier values, the 10 highest and 10 lowest values are excluded from the sorted list. The individual rest amplitude (${\text{amplitude}}_{\text{rest}}$) is calculated as the absolute difference between the mean of the 25 highest and the mean of the 25 lowest values from the remaining data. A correction factor is then calculated by dividing the expected amplitude by ${\text{amplitude}}_{\text{rest}}$, where the expected amplitude is set to 0.1:

$$\text{correction factor}=\frac{\text{expected Amplitude}}{\left|{\text{mean}\mathrm{HBO}}_{\text{restTop}25}-{\text{mean}\mathrm{HBO}}_{\text{restLo}25}\right|}.$$ (1)

The correction factor is used throughout the acquisition to correct the task amplitude with the rest amplitude.

Then, for each task phase, the feedback value for each i’th time-point is computed as

$${\text{feedback}}^{i}=(\text{mean}{\mathrm{HbO}}_{\mathrm{reg}}^{i}-\text{mean}{\mathrm{HbO}}_{\text{rest})}*\text{correction factor}$$

where $\text{mean}{\mathrm{HbO}}_{\mathrm{reg}}^{i}$ is the output of each i’th time-window during the regulation phase, $\text{mean}{\mathrm{HbO}}_{\text{rest}}$ is the 5-s average of the end of the previous rest phase, and the correction factor is explained earlier.

An expected HbO range of [−0.4,0.4] and a thermometer range of [0, 1] are defined in *MovAvg_SS.m.* To scale the expected HbO range to the thermometer range, the following equation is used:

$$r=\frac{\left({\text{feedback}-\mathrm{HBO}}_{\min})*({\text{thermo}}_{\max}{-\text{thermo}}_{\min}\right)}{{\mathrm{HBO}}_{\max}{-\mathrm{HBO}}_{\min}}+{\text{thermo}}_{\min}.$$ (2)

For example, if the calculated feedback is 0.24, the thermometer value would be 0.8 as $r=\frac{\left(0.24-(-0.4))*(1-0\right)}{0.4-(-0.4)}+0$. This results in 9 out of 10 thermometer bars being completely filled.

To find good values for the expected HbO range, it may be useful to observe NF amplitudes for the intended NF channels in pilot test runs before conducting the study.

#### 2.3.4 Practical steps

**Fig. 7:**
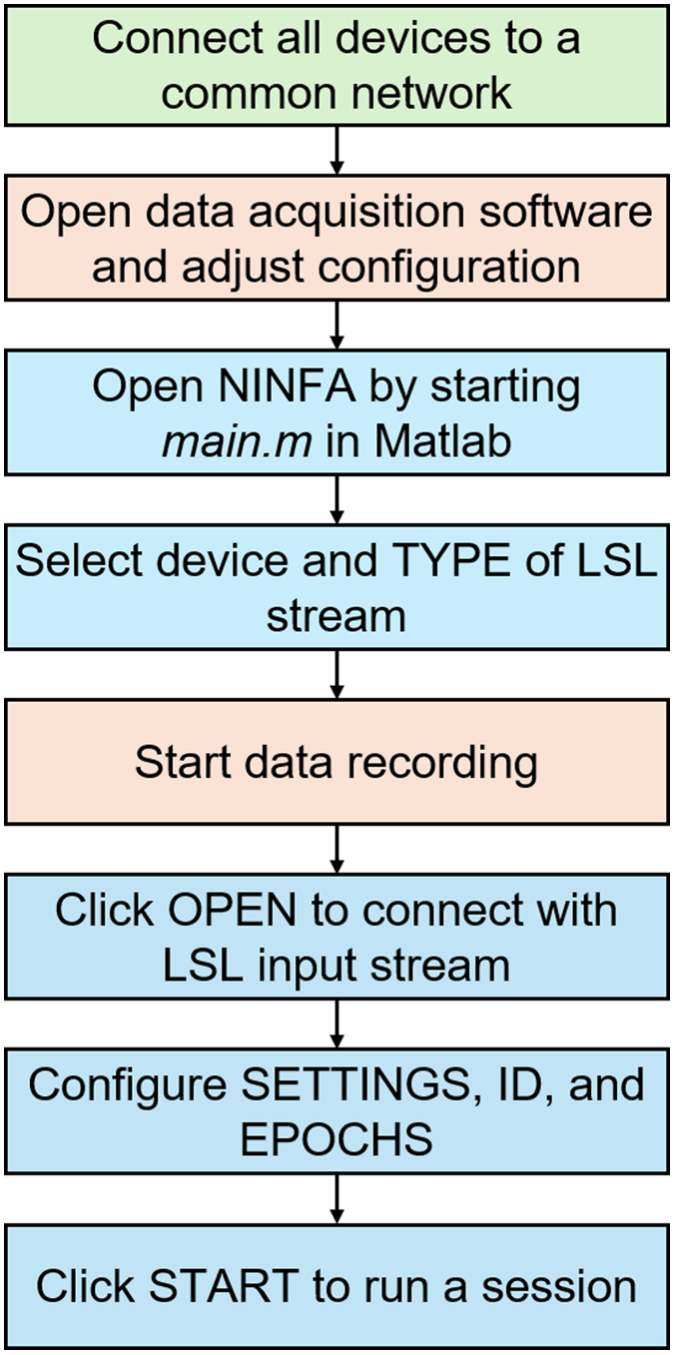
Flowchart of required steps for an assessment using the NINFA 1.2.0 application. The green box represents the very initial step, orange boxes represent steps related to the data acquisition software, and blue boxes represent steps related to the NINFA 1.2.0 application.

Once a device is defined and saved in the **devices** folder, the preprocessing and NF calculation functions are saved in the **protocols** folder, the experimental protocol parameters are stored in the **settings** folder, and data acquisition can begin. The various steps are explained in Figure 7. First, all devices must be connected to a common network, in our case the network of the NIRSport2 hardware. Then, the fNIRS montage is arranged on the participant’s head and selected accordingly in the fNIRS acquisition software. After opening the NINFA 1.2.0 interface using the *main.m* function, the recording can be initiated in the fNIRS software, and subsequently, the “Open” button in NINFA 1.2.0 can be clicked. By pressing the “File” button on the top-left side of the GUI, an already created file can be loaded to automatically change the Device, Setting, ID, and Epoch fields according to the stored information. Finally, with the “START” button, data acquisition can start. The acquisition is stopped promptly upon reaching the designated duration. If the user wants to stop the acquisition beforehand, this can be done by pressing the “STOP” button.

### 2.4 Output

**Fig. 8:**
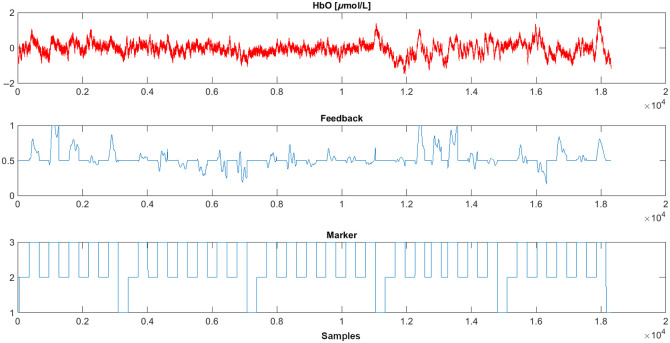
Protocol-specific exemplary output figure after a run through NINFA 1.2.0, showing the average of the NF channels, the feedback signal, as well as the marker ID (1 = baseline or break; 2 = rest phase; 3 = regulation phase).

The NINFA 1.2.0 application outputs both a figure and a.mat file. The .mat file is saved in the **sessions** folder, and it contains several variables: the sample frequency, the start and end time of the acquisition, the thermometer values, the marker values, and the raw data of the channels that were selected in the UI. Raw data consists of intensity data of the two-acquisition wavelengths (760 and 850 nm), and HbO and HbR concentration changes data. In our example protocol (*MovAvg_SS.m*), the output at the end of an assessment with NINFA 1.2.0 is configured to plot the averaged activity in the selected NF channels, NF values, and marker values (Figure 8 shows the results of one of our pilot subjects).

## 3 Discussion

In recent years, fNIRS has been gaining increasing attention in the field of NF and real-time acquisitions.^1^^–^^3^ However, freely accessible tools for working in this field and dealing with the state of the art are lacking. In this study, we present a new application, which in its current version can be used by researchers to perform not only real-time acquisitions mainly with fNIRS but also other modalities. The NINFA 1.2.0 application enables the acquisition of the signal in real time, its preprocessing, and visual feedback in the form of a thermometer that visualizes the preprocessed feedback signal. The version of the application presented here currently only has limited options in terms of signal preprocessing or the variability of the feedback display if no appropriate programming expertise is available but is currently being expanded.

We developed NINFA 1.2.0 because, according to our estimation, there is no freely available open-access tool for real-time NF with fNIRS, and, in addition, the compatibility of existing tools with different acquisition hardware and software is limited. Our idea was therefore to develop an application that offers the greatest possible flexibility in use and compatibility with different devices. The LSL connection offers maximum flexibility in terms of combination with different fNIRS acquisition software and hardware. Even the use of other modalities, such as EEG, is conceivable here. We have tested NINFA 1.2.0 using standard laptops and found no problems with real-time processing. The equipment needed to work with NINFA 1.2.0 therefore does not require any extraordinary requirements. The GUI for carrying out a measurement enables an intuitive application that does not require in-depth programming knowledge. However, creating new NF protocols requires adequate knowledge of the NF field and advanced programming skills. Although the current version of the application provides example code for real-time data preprocessing and visual feedback in the form of a thermometer, with appropriate programming skills, different preprocessing pipelines can be used, the existing pipeline can be supplemented, and additional sensory feedback modalities (e.g., auditory) or scenarios can be created. We would like to emphasize once again that we are only providing an example of how to calculate hemodynamic NF. It is not to be regarded as a comprehensive gold standard, and NINFA 1.2.0 is not the final version of the application.

There are a number of decisions that must be made before performing hemodynamic NF (but of course also with other imaging methods). First, the choice must be made whether HbO or HbR (or a combination of both) should be used as the feedback parameter. Although HbR is less susceptible to artifacts, the amplitudes are also lower than those of HbO, which in turn is more susceptible to artifacts.^36^ In the majority of NF studies, HbO has been chosen as the feedback parameter (most probably) due to the larger amplitudes,^39^ despite the lack of real-time correction to date.^3^ Visual feedback stands out as the most prevalent in existing literature^25^, yet alternative modalities such as auditory or haptic feedback are also feasible. In the current version, NINFA 1.2.0 only offers the visual display of the feedback signal in the form of a thermometer on different background colors. With appropriate expertise in programming, other visual types of feedback or auditory or haptic feedback can also be implemented.

The methods used for online data processing and artifact correction can also have a decisive influence on the calculation of the feedback signal.^40^ Here, we have provided some examples for real-time preprocessing (i.e., band-pass, Gauss low-pass, moving average filter, and SS regression). However, it is important to note that the optimal approach has not yet been established.

Not only the choice of the feedback parameter and the artifact correction but also the online feature extraction, i.e., the calculation of the feedback signal, can be carried out in different ways. In addition to the feedback of amplitudes in a single region of interest, functional connectivity measures can also be calculated and fed back.^40^

In our proposed protocol, each run ends with a break during which participants can move, talk, drink, and relax. To be able to observe the hemodynamic response in the last regulation phase of a run until it flattens out without artifacts, one would have to add another rest phase at the end of each run. In our specific case, we did not do this because the protocol is one that is to be used in a study with simultaneous tDCS, and thus, there are time constraints.

The toolbox is a dynamic, constantly evolving tool. We are currently further optimizing the GUI and some features. The application is currently based on MATLAB, and is therefore freely accessible, but a MATLAB license is required to use it. An implementation in Python is currently in progress, and then, the application can be used anywhere as a genuine open-access application without purchasing a license. Specifically, we are currently working primarily on setting up the Python version of the application. In addition to the open-access characteristics, this has the advantage that the Python-based MNE NIRS (https://github.com/mne-tools/mne-nirs/)^41^ provides a range of functions that could potentially be used for real-time correction.^22^ For instance, there are functions available for motion artifact correction (e.g., temporal derivative distribution repair)^42^ or temporal filtering. It should be noted that these have not been evaluated for real-time use, but for offline analysis. However, from our experience during the development of this first published version of the application, promising offline analysis methods such as Kalman filter and^34^ temporal embedded canonical correlation analysis^43^ can be tricky to implement in real time. We therefore need to test the implementation carefully. The very detailed documentation in the GitHub repository will ensure that users can easily cope with all the new features.

In the literature, one of the prevailing methods for mitigating physiological noise involves the utilization of short channels.^44^ In our first attempts at solving this problem, a limited number of samples made the short channel regression inconsistent by significantly degrading the signal. The first approach to reduce the influence of physiological noise on the signal for NINFA 1.2.0 was therefore to apply appropriate filters. Filters can be causal or acausal. The output of a causal filter depends only on past and present samples, whereas the output of an acausal filter also uses information from future samples. An acausal filter is applied to a data sequence, takes the output, reverses it, and applies the same filter again. The phase delays in forward and reverse filtering cancel each other out, so there is no effective filter delay.^45^ Despite the risk of phase shifts and distortions, any filter operating in real time must be a causal filter in the sense that its output can only depend on events that have already occurred and not on events that have not yet been measured.^45^ Although there are some limitations, several types of filters can be used in a real-time acquisition, such as the exponential moving average filter and the simple moving average filter.^21^ In addition, the RBJ biquad, Butterworth, Chebyshev, Elliptic, Bessel, and Legendre filters could be implemented.^46^ Few studies also reported the use of a Gauss low-pass filter.^47^^–^^49^

There are other parameters in the preprocessing pipeline that need to be defined, are subject to a certain degree of variability, and can certainly have an influence on the result of the preprocessing. Among the user inputs required before acquisition is the length of the sliding window. Within this window, the signal is preprocessed and analyzed for each sample to generate feedback in real time. A window that is too short can cause problems in preprocessing (due to the few samples) as the filter may not have enough data to function reliably. On the other hand, a window that is too long can be computationally more demanding during preprocessing and may compromise the accuracy of real-time NF by blending current information with past instances. Here, we propose a 5-s window as a good compromise. Of course, the window length must be chosen taking into account the sampling frequency, which determines the number of samples per second and therefore within the selected sliding window.

Another parameter that differs between acquisition devices and modalities is the sampling frequency. A higher sampling frequency obviously results in a larger number of samples within the same time interval. Consequently, a shorter time window can be chosen for the same number of samples, allowing the feedback to be calculated in a narrower time window. However, dark noise increases with higher sampling frequencies. The dark noise measure serves to characterize a detector’s sensitivity by assessing the amplitude and variance of its readings in the absence of input, typically under conditions of zero-incident light intensity.^50^ Elevated levels of dark noise may arise due to interference from ambient light sources, thereby signaling the necessity for immediate action to mitigate such noise. Remedial measures may include deactivating light sources, and employing shielding or overcaps, among others. The highest possible sampling frequency we could choose in our setup without increasing the dark noise too much was 10.2 Hz.

Another key challenge we encountered was the extensively reported variability of the neuronal response among subjects despite identical experimental conditions.^51^ In addition, each participant may exhibit a more or less pronounced physiological component within the signal. We found that one way to solve this problem is to use the individual resting state signal amplitude (min and max) of each participant during the rest phases preceding a regulation phase (specifically 15 s before the start of the regulation phase) and use this information to normalize the signal during the regulation phase. This allows the thermometer to show approximately the same deflections for each participant and not to show hardly any movement for subjects with a small amplitude range, whereas it fluctuates from one extreme to the other for subjects with greater variability. This normalization procedure not only corrects for between-subject variability but also for within-subject variability across a session. Further, this correction factor helps to deal with variations of the hemodynamic response function across brain regions.

We reported on an example of real-time fNIRS acquisition using the prefrontal cortex as the target region, which is a common target for fNIRS NF.^3^ This is not surprising as fNIRS is only suitable for measuring cortical areas,^11^ and the PFC plays an important role in many different functions, such as executive functions or the processing of emotions,^52^ and also the possibility of hair interference in this region is least likely.^20^ Participants were asked to use strategies that trigger a hemodynamic response in the prefrontal cortex. Our selective samples, consisting of psychologists, physicians, and cognitive scientists, have a good prior knowledge of prefrontal activation strategies, and this type of NF has been repeatedly shown to be feasible with fNIRS. Of course, it is possible to use other cortical brain areas to extract the NF signal using the application. Although activation of motor areas via motor imagery, which we used in the earlier versions of the application, is relatively easy to achieve, voluntary activation of frontal areas is more difficult because it is less clear which strategies can be used successfully. In addition, it may be easier to stop the movement (imagery) after completing the task phase than to turn down the activation of the frontal areas during the rest phase. Hence, when changing the target region, it is important to consider the following: the possible different signal amplitudes among brain regions, the variability among subjects, and the difference between regulation and rest. The problem of signal amplitude can be overcome by setting the normalization parameters. The thermometer range must be defined by the user for each target region before starting a study. However, the signal variability among subjects is automatically mitigated based on the signal during rest phases (see above).

## 4 Conclusion

With NINFA 1.2.0, we present a publicly available, user-friendly, and flexible application for real-time fNIRS NF applications. We describe the equipment, the workflow of data acquisition, processing, and presentation, which ensures good and uncomplicated interaction with the various tools. We tested the stability and reliability of the computational performance of preprocessing and analysis methods. NINFA 1.2.0 is intended as an application that can, should, and may continue to evolve with input from the community.

## Supplementary Material

10.1117/1.NPh.12.2.026601.s01

## Data Availability

The application is publicly available at https://github.com/PsychoOI/NINFA.
