# Supplementary material for: NINFA: Non-commercial interface for neuro-feedback acquisitions
Source: Neurophotonics. 2025 May 14;12(2):026601. doi: 10.1117/1.NPh.12.2.026601 (PMC12077575; doi:10.1117/1.NPh.12.2.026601)
Supplement: Supplementary file 1 [file NPh_012_026601_SD001.pdf]

Practical Recommendations

In this section, we share our experiences and challenges while testing various real-time methods for neurofeedback. First, we began to test different possibilities of real-time correction using simulations. For the sophisticated GLM method, with the small amount of real-time data, we were unable to estimate the contribution of short-separation (SS) channels, leading to an inadequate and highly variable result within the framework of the simulations. The Kalman filter uses the observed values of the time series and estimates the trend and noise in the time series and combines them to produce a smoothed time series. For the Kalman filter we did not manage to achieve meaningful tuning parameters. Therefore, we tested common filters, such as a lowpass filter (0.2 Hz; 0.3 Hz), bandpass filter, as well as the Gauss low-pass filter (15th order) with a cutoff frequency of 0.022 Hz [37–39] or a bandstop filter (0.2-0.4 Hz; 0.08-0.9 Hz) with different baseline lengths (between 5 and 10 seconds) and "positions" (15-25 s; 20-30 s; 25-30 s), but also different sliding window sizes (5 s, 10 s) and sample rates (10.2 Hz; 30.52 Hz; 40.7 Hz). Our main problem was that some participants had extremely large-amplitude slow oscillations that withstood all filters. In the feedback, the large fluctuations resulted in the signal being uncontrollable. This led to the decision to introduce an individual scaling factor based on the amplitude range in the baseline phase in order to make the movement range of the thermometer roughly the same for each participant. This, in combination with the Gaussian filter, produced the best results when not applying SS correction. As expected, the tests with motor execution produced large and reliable amplitudes for all participants and were therefore a good basis for a sanity check of the code (e.g., what if motor execution is carried out in the rest phase while relaxation occurs in the task phase? (lower HbO concentration expected in the task phase compared to the baseline) or if relaxation occurs throughout the entire experiment? (random fluctuations expected)). Motor imagery is another reliable option for testing and the results from the motor execution phase were confirmed. As expected, we observed smaller amplitudes for our neurofeedback tests on prefrontal hemodynamics, and there were more runs in which the regulation in the desired direction did not succeed. However, the regulation of the prefrontal cortex in neurofeedback is inherently challenging, as it is less accessible in everyday life for participants. At the end, through careful adjustment of parameters, we were able to achieve reliable results. We then updated our approach by incorporating short-separation (SS) regression. Klein (2024) provides an excellent explanation of various approaches for real-time physiological correction in the fNIRS signal. Since SS regression effectively removes physiological noise, we selected it as the optimal approach for the main manuscript. Finally, although the Gaussian filter is not widely used in the literature, we found it to be an interesting approach and believe it could be considered for future studies.
